# Isorhynchophylline ameliorates stress-induced emotional disorder and cognitive impairment with modulation of NMDA receptors

**DOI:** 10.3389/fnins.2022.1071068

**Published:** 2022-12-15

**Authors:** Chen Wang, Ming-Hao Zhu, Na Sun, Wei Shen, Ning Jiang, Qin-Shi Zhao, Yong-Xiang Zhang, Yan Huang, Wen-Xia Zhou

**Affiliations:** ^1^State Key Laboratory of Toxicology and Medical Countermeasures, Beijing Institute of Pharmacology and Toxicology, Beijing, China; ^2^State Key Laboratory of Phytochemistry and Plant Resources in West China, Kunming Institute of Botany, Chinese Academy of Sciences (CAS), Kunming, China

**Keywords:** isorhynchophylline (PubChem CID: 3037048), corticosterone, long-term potentiation, chronic unpredictable mild stress, D-serine, NMDA receptors

## Abstract

**Introduction:**

Isorhynchophylline is one of the main active ingredients from *Uncaria rhynchophylla*, the effects and mechanisms of isorhynchophylline on stress-induced emotional disorders and cognitive impairment remain unclear.

**Methods:**

Long-term potentiation (LTP) *in vivo* was used for synaptic plasticity evaluation; chronic unpredictable mild stress (CUMS) model was used to evaluate the effect of isorhynchophylline on stress induced emotional disorders and cognitive impairment; sucrose preference test (SPT), open field test (OFT), and elevated plus maze (EPM) were used to evaluate emotional disorders; morris water maze (MWM) test was used to evaluate cognitive impairment; Western blotting (WB) was used to the expression of proteins; high performance liquid chromatography (HPLC) was used to quantify neurotransmitters; Nissl staining was used to identify pathological changes induced by stress.

**Results:**

In this study, we found that isorhynchophylline improved corticosterone-induced *in vivo* LTP impairment significantly, indicating positive effects on stress. Therefore, 28-day CUMS model was adopted to evaluate the anti-stress effects of isorhynchophylline. The results showed that isorhynchophylline improved CUMS-induced weight loss, anxiety- and depression-like behaviors, and spatial memory impairment. Isorhynchophylline reduced CUMS-induced corticosterone elevation. N-methyl-D-aspartic acid (NMDA) receptors play an important role in the process of emotion and memory. Glutamate and the expression of GluN2B increased in the CUMS mice, while D-serine and the expression of serine racemase (SR) decreased significantly, and isorhynchophylline restored these changes to normal level.

**Conclusion:**

These results indicated that isorhynchophylline ameliorated stress-induced emotional disorders and cognitive impairment, modulating NMDA receptors might be one of the underlying mechanisms.

## 1 Introduction

Stress is broadly defined as an anticipated disruption of homeostasis or a threat to wellbeing (Kim and Diamond, 2002; McEwen, 2007; Ulrich-Lai and Herman, 2009). A number of stress-associated diseases have been identified in humans, such as hypertension, diabetes, gastric-intestinal ulceration (Kim et al., 2006). High-intensity stress could not only cause anxiety and depression symptoms (Willner, 2017; Lupien et al., 2018), but also increase the risk of various neurological disorders (McEwen, 2000; Radahmadi et al., 2014). Alleviating the symptoms like anxiety and depression is the primary treatment in clinic, the commonly used drugs are serotonin reuptake inhibitors, such as sertraline and fluoxetine (Giacomini et al., 2016; Noorafshan et al., 2019; Rauch et al., 2019). Recently, the N-methyl-D-aspartate (NMDA) receptors antagonist ketamine or its S (+)-isomer has been used for the treatment of stress-induced depressive disorders and post-traumatic stress disorder (PTSD) in both humans and animals (Feder et al., 2014; Brachman et al., 2016; McGowan et al., 2018; Wang et al., 2022). However, the current drugs could only alleviate stress-induced emotional disorders like anxiety and depression with little improvement on stress-induced cognitive impairment, and these drugs might even cause cognitive impairment in long-term use (Luo et al., 2021).

Isorhynchophylline is one of the main active ingredients from *Uncaria rhynchophylla*. *Uncaria rhynchophylla*, a traditional Chinese medicine, which is mainly used to treat cardiovascular and central nervous systems diseases, such as lightheadedness, convulsions, numbness, and hypertension (Shi et al., 2003). Modern pharmacological researches reveal that isorhynchophylline has multiple neural protective effects. Isorhynchophylline could improve cognitive impairment in Alzheimer’s disease (Xian et al., 2014a), D-galactose (Xian et al., 2014b), aluminum chloride (Li et al., 2018) and ischemia (Kang et al., 2004) animal models. Isorhynchophylline could also ameliorate neural plasticity deficits caused by many factors, such as ischemia, D-galactose and aluminum chloride (Kang et al., 2004; Xian et al., 2014b; Li et al., 2018). Reducing beta-amyloid peptide (Aβ)-induced neurotoxicity, neuronal apoptosis and tau protein hyperphosphorylation (Xian et al., 2012, Xian et al., 2013, 2014a) contributed to isorhynchophylline’s neural protective effects. NMDA and 5-HT_2_ receptors (Kang et al., 2002, 2004) were the potential targets for isorhynchophylline. Recently, it has reported that isorhynchophylline had antidepressant-like effects (Xian et al., 2017, 2019). These results indicate that isorhynchophylline has potential effects on stress-induced emotional disorders and cognitive impairment. Thus, this study is to investigate whether isorhynchophylline has protective effect against stress-induced emotional disorders and cognitive impairment.

Corticosterone is both the biomarker and effector molecule of stress in rodents. Excess corticosterone could cause cognitive (Dominguez et al., 2019) and neural plasticity impairments (Wang et al., 2021; Huang et al., 2022). Therefore, corticosterone induced long-term potentiation (LTP) impairment was used for preliminary evaluation of isorhynchophylline. Then the 28-day chronic unpredictable mild stress (CUMS) model was adopted to confirm whether isorhynchophylline could alleviate emotional disorders and cognitive impairment, and finally observing the effect of isorhynchophylline on CUMS-induced dysfunction of NMDA receptor.

## 2 Materials and methods

### 2.1 Animals and drug treatment

Two-month-old male C57BL/6J mice, which is commonly used for CUMS model (Lu et al., 2019), were purchased from Beijing Vital River Laboratory Animal Technology Co., Ltd. Mice were kept in plastic cages and allowed free access to food and water under standard housing conditions (room temperature 23 ± 1°C and humidity of 55 ± 5%) with a 12-h light/12-h dark cycle. Experiments started after 7 days acclimating to the laboratory environment. The Institute of Animal Care and Use Committee of the National Beijing Center for Drug Safety Evaluation and Research approved all the experiments (No.: IACUC-DWZX-2021-590).

For *in vivo* electrophysiology, 50 mg/kg corticosterone (CORT, TCIchemicals, Shanghai, China) was used to mimic stress 60 min before high frequency stimulation (HFS). Both intracerebroventricular and intragastric administration were used in this study. Intracerebroventricular administration only used for preliminary evaluation; intragastric administration, which is like clinical routine, was used for further evaluation. For intracerebroventricular administration, 2 μg per animal (isorhynchophylline) was used; For intragastric administration, 20/40/80 mg/kg isorhynchophylline was used.

For intracerebroventricular injection, the coordinates are: −0.22 mm from Bregma, 1 mm lateral to the sagittal suture and 2.5 mm deep from the skull surface, according to *Mouse Brain in Stereotaxic Coordinates* (Franklin and Paxinos, 2008). A syringe pump, at a rate of 1 μl/min, was used to deliver the drug into the right lateral ventricle. The needle was left in place for 1 min after discontinuation of plunger movement to prevent backflow.

For CUMS, mice were separated into three groups, which were Control group (Control), chronic unpredictable mild stress group (CUMS) and chronic unpredictable mild stress + isorhynchophylline group (Iso). Isorhynchophylline was intragastric administered 20 mg/kg/day from the −7 to 28 d. Mice in Control and CUMS groups were given equal volumes of 0.5% sodium carboxymethyl cellulose (Sigma-Aldrich Corporation).

### 2.2 *In vivo* LTP recording

*In vivo* LTP recording was conducted as previously described (Huang et al., 2013). Mice were anesthetized with pentobarbital sodium,then fitted with ear cuffs and placed in a stereotaxic frame. A stimulating electrode (stainless steel bipolar) was placed in the perforant path (PP), the evoked potentials were recorded with a stainless-steel electrode in the cell body layer of dentate gyrus (DG). The WinLTP program^1^ was used to initiate the electrical stimulus and record the data. After obtaining a stable stimulus–response curve baseline at fixed current intensity, the current intensity was regulated to evoke a 1/3–1/2 maximum population spike (PS) amplitude. After recording the 30 min baseline, LTP was induced by high-frequency stimulus (HFS) (250 Hz, three trains 10 s apart, eight 0.1 ms pulses in each train), and PSs were recorded for another 60 min. The mean PS amplitudes during 0–30 min was normalized to 100% as baseline; the relative PS amplitudes (31–90 min) were normalized relative to the baseline period before HFS.

### 2.3 Chronic unpredictable mild stress procedure

According to the previous description (Willner, 2017; Liu M. Y. et al., 2018), the CUMS procedure was conducted for 28 days. CUMS-treated mice were subjected to 14 stressors with a random schedule. These stressors include food deprivation (24 h), water deprivation (24 h), cage tilt (24 h, 45°), dark during the day (12 h), lights on at night (12 h), damp bedding (24 h), no bedding (24 h), noise stimulation (1 h, 100 dB), electric shock (1 h, 0.8 mA, 5 s/min), tail pinch (1 min), tail suspension (30 min), cage shake (1 h, 220 r/min), restrained in tube (2 h) and cold water swimming (6 min, 10 °C), the detailed schedule is shown in Table 1.

**TABLE 1:** The protocol of 28 days chronic unpredictable mild stress.

Day week	Day 1	Day 2	Day 3	Day 4	Day 5	Day 6	Day 7
Week 1	LN	FD	CT	DB	DD	NB	WD
	RS	CW	TP	ES	TS	CS	NS
Week 2	NB	WD	DB	RS	DD	FD	DB
	CW	DD	TS	TP	CW	CT	CS
Week 3	NB	WD	RS	CT	DD	FD	LN
	TS	NB	NS	ES	TP	CT	TS
Week 4	DD	WD	CT	DB	FD	LN	WD
	CW	ES	CS	RS	TP	TS	NS

FD, food deprivation (24 h); WD, water deprivation (24 h); CT, cage tilt (24 h, 45°); DD, dark during the day (12 h); LN, lights on at night (12 h); DB, damp bedding (24 h); NB, no bedding (24 h); NS, noise stimulation (1 h, 100 dB); ES, electric shock (1 h, 0.8 mA, 5 s/min), TP, tail pinch (1 min), TS, tail suspension (30 min); CS, cage shake (1 h, 220r/min); RS, restrained in tube (2 h); CW, cold water swimming (6 min, 10°C).

### 2.4 Behavior tests

#### 2.4.1 Sucrose preference test (SPT)

The SPT was according to previous description (Liu M. Y. et al., 2018). Four days before CUMS, all animals were habituated to drink sucrose solution (1%, w/v) by replacing normal water for 2 days (48 h). The position of the bottles was changed several times during the period. During test, all mice were deprived of food and water for 24 h, starting at 10 a.m. 24 h later. Each animal was provided with 1% sucrose solution and normal water individually for 1 h, and the weights of sucrose solution and water consumed were recorded accordingly.

#### 2.4.2 Open field test

The OFT was performed as described previously (Huang et al., 2017). The arena was partitioned into peripheral area and central area. After 30 min acclimation in the test room, mice were allowed to explore the open field for 5 min and then they were returned to home age. Central area duration and visits were recorded as indicators of anxiety and exploratory behaviors.

#### 2.4.3 Elevated plus maze (EPM)

The elevated plus-maze apparatus was elevated 100 cm above the floor. The maze consisted of two open arms (50 cm × 10 cm) and two closed arms (50 cm × 10 cm × 10 cm) joined by a central square (10 cm × 10 cm). mice were put in the central square and allowed to explore for 5 min, then they were returned to home age.

#### 2.4.4 Morris water maze (MWM) test

The MWM test was performed as described previously (Vorhees and Williams, 2006). The learning phase contained four trials per day for five consecutive days and the probe trial was carried out on the sixth day. During the learning phase, mice were allowed to find the platform for 60 s, if the mouse failed to find the platform on time, it was guided to the platform and remained on the platform for 15 s. During the probe trial phase, mice were given a 60 s exploring period in the pool without platform, the latency and time in the target quadrant were regarded as indicators of memory.

The order of these tests is: Sucrose Preference Test, Open Field Test, Elevated plus Maze, Morris Water Maze Test.

### 2.5 Corticosterone evaluation

Plasma corticosterone was measured by an enzyme-linked immunosorbent assay kit (Cloud-Clone Corp, USA) according to the Instruction manual.

### 2.6 Western blotting (WB)

Western blotting was performed as described previously with minor modifications (Taylor and Posch, 2014). Ten mg hippocampal samples were homogenized in 0.1 ml lysis buffer and then centrifuged at 15,000 g for 15 min at 4°C. The concentrations of protein were determined by Bradford protein assay kit (PR102, Galen Biopharm International Co., Ltd.). Equal amounts of protein were boiled in loading buffer (100 mM Tris–HCl of pH 6.8, 4% sodium dodecyl sulfate, 200 mM DTT, 0.2% bromophenol blue, and 20% glycerol) for 5 min before loading on a SDS polyacrylamide gel. Electrophoresis was performed at 60 V for 30 min and then 100 V for 90 min, followed by wet transfer onto a nitrocellulose membrane at 100 V for 60 min. The membrane was blocked for 60 min in blocking solution (5% non-fat dry milk, 0.05% Tween-20, phosphate buffered saline) and then incubated at 4°C overnight with rabbit anti-SR antibody (1:1000, Sigma-Aldrich) and rabbit anti-NR2A/B antibody (1:1,000, Sigma-Aldrich). After 30 min washes with 0.05% Tween-20, PBS, the primary antibodies were detected with the horseradish peroxidase-conjugated secondary antibodies and chemiluminescent HRP substrate (Thermo Fisher Scientific Inc., Waltham, MA, USA). Band density values were normalized to β-actin.

### 2.7 High performance liquid chromatography (HPLC)

HPLC was performed as described previously with minor modifications (Grant et al., 2006). Hippocampal samples were homogenized in methanol and centrifuged for 15 min at 15,000 g at 4°C. The calculated amount of OPA was dissolved in 5 mL of methanol in a volumetric flask and diluted to the mark with a borate buffer solution (PH = 9.3). The sample was derivatizated for 35 min before loading sample. The mobile phase contains a buffered solution containing 0.1 M NaH_2_PO_4_, 20 m 0.1 mM Na_2_EDTA and its pH was adjusted to 5.8 with phosphoric acid. The flow rate was 0.75 ml/min, the detector potential was + 0.75 V with respect to the calomel reference electrode and the sensitivity was set at 50 nA full-scale detection. Chromatographic column is an Agilent reversed-phase chromatographic column (C-18, 4.6 mm × 250 mm, 5 μm).

### 2.8 Nissl staining

The mice brains were removed after anesthesia and fixed in 4% paraformaldehyde. The sections (4 μm) were stained using 0.5% cresyl violet acetate (Beyotime, China). The integrated optical density (IOD) of Nissl bodies was quantified by using Image J software (Version 1.48, National Institutes of Health).

### 2.9 Statistical analysis

The data are presented as the mean ± SEM. GraphPad Prism 6.0 (Inc., La Jolla, CA, USA) was used to plot and analyze the data. Student’s *t*-test was used to analyze two groups and one-way analysis of variance (ANOVA) followed by Dunnett’s multiple comparisons test was used when comparing more than two groups. A two-way repeated measures ANOVA was adopted to analyze the changes of body weight during CUMS and MWM escape latency during learning phase. *P* < 0.05 was considered as statistically significant.

## 3 Results

### 3.1 Effects of isorhynchophylline on hippocampal LTP in corticosterone-treated mice

**FIGURE 1:**
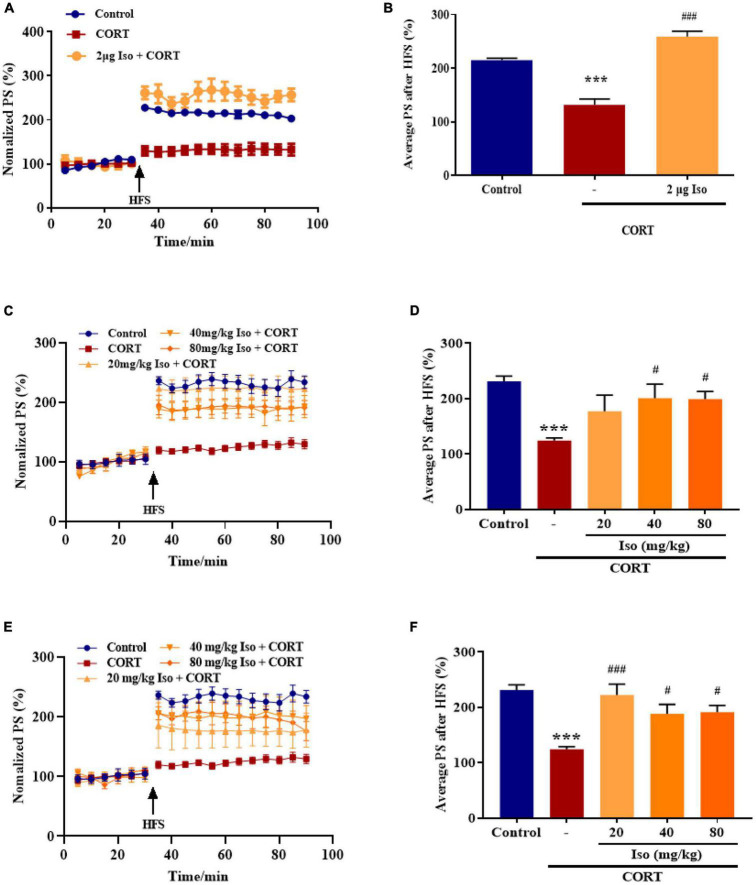
Effects of isorhynchophylline on hippocampal LTP in corticosterone (CORT)-treated mice. **(A)** The time course of average relative PS amplitudes. **(B)** The average relative PS amplitudes post-HFS (31–90 min). Isorhynchophylline (2 μg, i.c.v) significantly improved CORT-induced LTP impairment. CORT/vehicle was injected 60 min before HFS and isorhynchophylline was administrated 30 min before CORT/vehicle. **(C)** The time course of average relative PS amplitudes. **(D)** The average relative PS amplitudes post-HFS (31–90 min). Isorhynchophylline (40/80 mg/kg, i.g.) significantly improved CORT-induced LTP impairment. **(E)** The time course of average relative PS amplitudes. **(F)** The average relative PS amplitudes post-HFS (31–90 min). Isorhynchophylline (20/40/80 mg/kg for seven days, i.g.) significantly improved CORT-induced LTP impairment. The data are presented as mean ± SEM. ***P < 0.001 compared to the Control group; ^#^*P* < 0.05, ^###^*P* < 0.001 compared to the corticosterone group.

The average values of the relative PS amplitudes in CORT-treated mice were significantly lower than that in the control mice, while isorhynchophylline (2 μg, i.c.v.) significantly reverse the decreased PS amplitudes (one-way ANOVA followed by Dunnett’s tests; *F_2_,_12_* = 52.66, *P* < 0.001; Cort *vs.* control: *P* < 0.001; 2 μg Iso *vs.* Cort: *P* < 0.001; *n* = 5; Figures 1A, B).

Subsequently, we observed the effect of single administration of isorhynchophylline (20/40/80 mg/kg, i.g.) on corticosterone-induced LTP impairment. Results showed the average values of the relative PS amplitudes in CORT-treated mice were significantly lower than that in the control mice, while 40 and 80 kg/mg isorhynchophylline significantly reverse the decreased PS amplitudes and 20 mg/kg isorhynchophylline showed no effect (one-way ANOVA followed by Dunnett’s tests; *F_4_,_20_* = 6.204, *P* = 0.002; Cort *vs.* control: *P* < 0.001; 20 mg/kg Iso *vs.* Cort: *P* = 0.105; 40 mg/kg Iso *vs.* Cort: *P* = 0.013; 80 mg/kg Iso *vs.* Cort: *P* = 0.014; n = 5; Figures 1C, D).

Then, we observed the effect of administration of isorhynchophylline (20/40/80 mg/kg, i.g.) for 7 consecutive days on corticosterone-induced LTP impairment. Results showed that 20, 40 and 80 kg/mg isorhynchophylline significantly reverse the decreased PS amplitudes by corticosterone (one-way ANOVA followed by Dunnett’s tests; *F_4_,_20_* = 9.177, *P* < 0.001; Cort *vs.* control: *P* < 0.001; 20 mg/kg Iso *vs.* Cort: *P* < 0.001; 40 mg/kg Iso *vs.* Cort: *P* = 0.026; 80 mg/kg Iso *vs.* Cort: *P* = 0.021; *n* = 5; Figures 1E, F).

These results showed that both central and peripheral administration of isorhynchophylline significantly improved corticosterone-induced LTP impairment, indicating potential effects on stress.

### 3.2 Effects of isorhynchophylline on emotional disorder in CUMS mice

**FIGURE 2:**
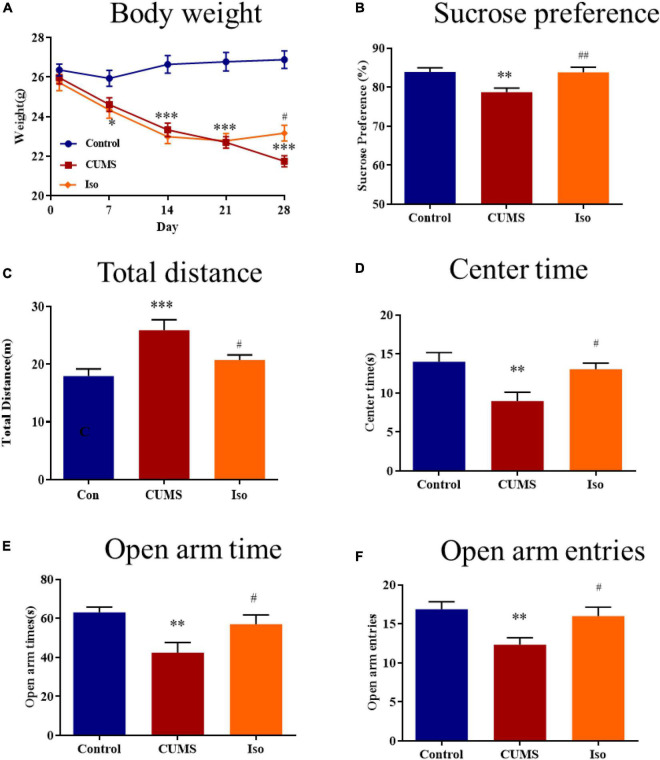
Effects of isorhynchophylline on body weight, sucrose preference, open field test and elevated plus maze in CUMS mice. **(A)** Isorhynchophylline significantly alleviated CUMS-induced body weight loss. **(B)** Isorhynchophylline significantly increased sucrose preference index in CUMS mice. **(C)** Isorhynchophylline significantly decreased total distance in CUMS mice. **(D)** Isorhynchophylline significantly increased central retention time in CUMS mice. **(E)** Isorhynchophylline significantly increased open arm times in CUMS mice. **(F)** Isorhynchophylline significantly open arm entries in CUMS mice. The data are presented as mean ± SEM. ^**^P < 0.01, ^***^P < 0.001 compared to the Con group; ^#^*P* < 0.05, ^##^*P* < 0.01 compared to the CUMS group.

The body weight in the control group kept increasing stably; the body weight in the CUMS group was significantly lower than that of the control group on the 7th, 14th, 21st, and 28th day, and isorhynchophylline improved the body weights on the 28th day (two-way repeated measures ANOVA followed by Dunnett’s tests; Time: *P* < 0.001; Treatment: *P* < 0.001; Day 1: CUMS *vs.* control: *P* = 0.578; Iso *vs.* CUMS: *P* = 0.856; Day 7: CUMS *vs.* control: *P* = 0.039; Iso *vs.* CUMS: *P* = 0.837; Day 14: CUMS *vs.* control: *P* < 0.001; Iso *vs.* CUMS: *P* = 0.708; Day 21: CUMS *vs.* control: *P* < 0.001; Iso *vs.* CUMS: *P* = 0.979; Day 28: CUMS *vs.* control: *P* < 0.001; Iso *vs.* CUMS: *P* = 0.015; *n* = 10; Figure 2A).

For SPT test, the results showed the sucrose preference index of mice in the CUMS group was significantly lower than that in the control group, and isorhynchophylline improved the sucrose preference indexes (one-way ANOVA followed by Dunnett’s tests; *F_2_,_27_* = 8.566, *P* = 0.001; CUMS *vs.* control: *P* = 0.003; Iso *vs.* CUMS: *P* = 0.002; *n* = 10; Figure 2B).

For OFT test, the results showed that the total distance increased in CUMS group significantly, while the central retention time is higher than that of the CUMS group, and isorhynchophylline improved theses changes in OFT (one-way ANOVA followed by Dunnett’s tests; For total distance: *F_2_,_27_* = 8.784, *P* = 0.001; CUMS *vs.* control: *P* < 0.001; Iso *vs.* CUMS: *P* = 0.022; For central retention time: *F_2_,_27_* = 7.509, *P* = 0.003; CUMS *vs.* control: *P* = 0.002; Iso *vs.* CUMS: *P* = 0.013; *n* = 10; Figures 2C, D).

For EPM test, the results showed that the open arm time and open arm entries were significantly decreased in CUMS group, and isorhynchophylline improved theses changes in EPM (one-way ANOVA followed by Dunnett’s tests; For open arm time: *F_2_,_27_* = 6.655, *P* = 0.005; CUMS *vs.* control: *P* = 0.003; Iso *vs.* CUMS: *P* = 0.029; For open arm entries: *F_2_,_27_* = 6.401, *P* = 0.005; CUMS *vs.* control: *P* = 0.004; Iso *vs.* CUMS: *P* = 0.021; *n* = 10; Figures 2E, F).

These results suggested that isorhynchophylline effectively alleviated anxiety- and depression- like behaviors of mice caused by CUMS.

### 3.3 Effects of isorhynchophylline on the spatial memory in CUMS mice

**FIGURE 3:**
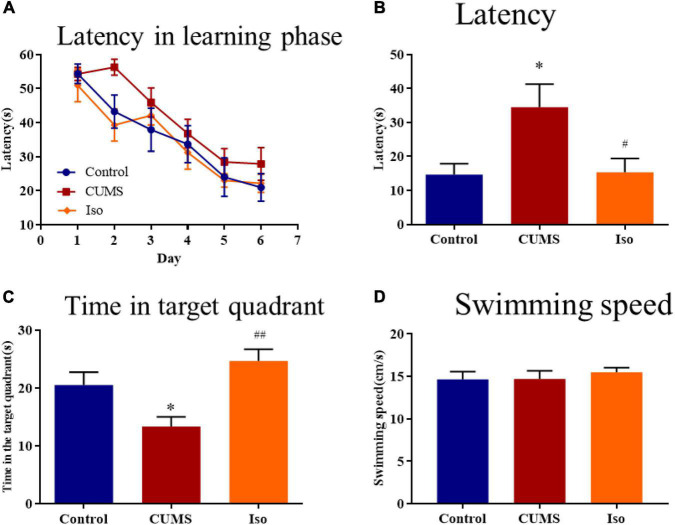
Effects of isorhynchophylline on the spatial memory in MWM test in CUMS mice. **(A)** The escape latency of mice in learning phase. **(B)** The escape latency in probe trial was significantly decreased by isorhynchophylline in CUMS mice. **(C)** The time spent in the target quadrant was significantly increased by isorhynchophylline in CUMS mice. **(D)** There was no difference in swimming speed in each group. The data are presented as mean ± SEM. *P < 0.05 compared to the con group; ^ #^*P* < 0.05, ^ ##^*P* < 0.01 compared to the CUMS group.

During the learning phase, the escape latency of mice in each group showed a downward trend and there was no statistical difference among groups (Figure 3A). During the probe phase, the escape latency in the CUMS group was significantly longer than that in the control group, and isorhynchophylline improved the latency significantly (one-way ANOVA followed by Dunnett’s tests; *F_2_,_27_* = 5.770, *P* = 0.008; CUMS *vs.* control: *P* = 0.011; Iso *vs.* CUMS: *P* = 0.014; *n* = 10; Figure 3B). The time in the target quadrant of the CUMS group was significantly shorter than that of the control group, and isorhynchophylline improved the time in the target quadrant significantly (one-way ANOVA followed by Dunnett’s tests; *F_2_,_27_* = 7.769, *P* = 0.002; CUMS *vs.* control: *P* = 0.045; Iso *vs.* CUMS: *P* = 0.001; *n* = 10; Figure 3C). There was no difference in the swimming speed of the mice in each group during the probe phase (Figure 3D).

The above results suggested that isorhynchophylline effectively improved the spatial memory deficit in mice caused by CUMS.

### 3.4 The mechanisms of isorhynchophylline on CUMS mice

**FIGURE 4:**
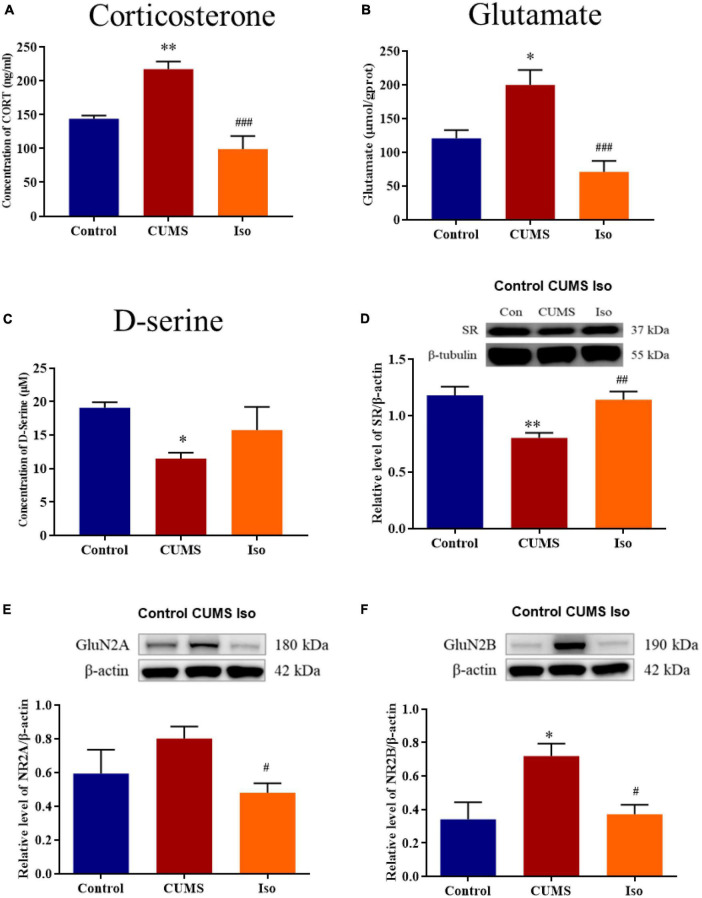
Effects of isorhynchophylline on plasma corticosterone, glutamate, SR, D-serine, GluN2A and GluN2B in CUMS mice. **(A)** Isorhynchophylline significantly decreased the serum corticosterone level in CUMS mice. **(B)** Isorhynchophylline significantly decreased the hippocampal glutamate level in CUMS mice. **(C)** Isorhynchophylline increased the level of D-serine in CUMS mice. **(D)** Isorhynchophylline significantly increased the expression of SR in CUMS mice. **(E)** Isorhynchophylline significantly decreased the expression of GluN2A in CUMS mice. **(F)** Isorhynchophylline significantly decreased the expression of GluN2B in CUMS mice. The data are presented as mean ± SEM. *P < 0.05, ^**^P < 0.01 compared to the Con group; ^ #^*P* < 0.05, ^ ##^*P* < 0.01, ^ ##^*P* < 0.001 compared to the CUMS group.

The plasma corticosterone in the CUMS group was significantly increased compared with control, and corticosterone in the isorhynchophylline group was significantly lower than that in the CUMS group (one-way ANOVA followed by Dunnett’s tests; *F_2_,_12_* = 13.16, *P* < 0.001; CUMS *vs.* control: *P* = 0.009; Iso *vs.* CUMS: *P* < 0.001; *n* = 5; Figure 4A). The hippocampus glutamate in the CUMS group increased compared with control, and the glutamate in the isorhynchophylline group was significantly lower than that in the CUMS group (one-way ANOVA followed by Dunnett’s tests; *F_2_,_12_* = 12.37, *P* = 0.001; CUMS *vs.* control: *P* = 0.011; Iso *vs.* CUMS: *P* < 0.001; *n* = 5; Figure 4B). The hippocampus D-serine, the NMDA receptors co-agonist, was significantly reduced in the CUMS group, and the content of D-serine in the isorhynchophylline group was higher than that of the CUMS group (one-way ANOVA followed by Dunnett’s tests; *F_2_,_12_* = 3.215, *P* = 0.076; CUMS *vs.* control: *P* = 0.048; Iso *vs.* CUMS: *P* = 0.299; *n* = 5; Figure 4C).

The expression of SR, a key synthetase of hippocampal D-serine, was significantly decreased in the CUMS group, while the expression of SR in the isorhynchophylline group was significantly higher than that in the CUMS group (one-way ANOVA followed by Dunnett’s tests; *F_2_,_12_* = 9.771, *P* = 0.003; CUMS *vs.* control: *P* = 0.003; Iso *vs.* CUMS: *P* = 0.007; *n* = 5; Figure 4D). The expression of hippocampal GluN2A in the CUMS group showed an increasing trend, and the expression of GluN2A in the hippocampus in the isorhynchophylline group was significantly lower than that of the CUMS group (one-way ANOVA followed by Dunnett’s tests; *F_2_,_12_* = 3.664, *P* = 0.057; CUMS *vs.* control: *P* = 0.171; Iso *vs.* CUMS: *P* = 0.038; *n* = 5; Figure 4E). The expression of GluN2B in the CUMS group was significantly higher than that in the control group, and the GluN2B expression in the isorhynchophylline group was significantly lower than that in the CUMS group (one-way ANOVA followed by Dunnett’s tests; *F_2_,_12_* = 6.32, *P* = 0.013; CUMS *vs.* control: *P* = 0.016; Iso *vs.* CUMS: *P* = 0.020; *n* = 5; Figure 4F).

**FIGURE 5:**
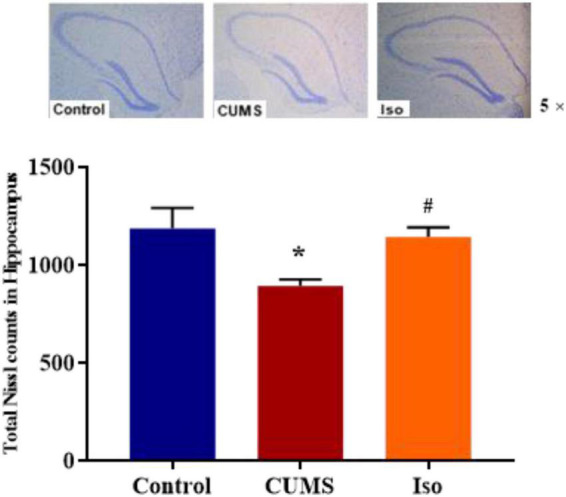
Effects of isorhynchophylline on Nissl staining in hippocampus. The data are presented as mean ± SEM. *P < 0.05 compared to the Con group; ^ #^*P* < 0.05 compared to the CUMS group.

Nissl staining results showed that the IOD of Nissl bodies was significantly lower in the CUMS group than that in the control group, and IOD of Nissl bodies in isorhynchophylline group was significantly higher than that in CUMS group (one-way ANOVA followed by Dunnett’s tests; *F_2_,_12_* = 5.230, *P* = 0.023; CUMS *vs.* control: *P* = 0.028; Iso *vs.* CUMS: *P* = 0.031; *n* = 5; Figure 5).

## 4 Discussion

When endogenous or exogenous environmental stimuli is perceived as aversive, or threatening, the systemic neuroendocrine response is activated, this process is called stress response (Lucassen et al., 2014). Stress can activate the HPA axis, and glucocorticoids are one of the main biomarkers for stress (Akirav, 2004; Mora et al., 2012). There are 2 types of receptors for glucocorticoids, the mineralocorticoid receptor (MR) and the glucocorticoid receptor (GR) (Reul and de Kloet, 1985). Both MR and GR expressed in hippocampus (Reul and De Kloet, 1986), and related to stress-induced cognition impairment and emotional disorders. Stress or glucocorticoids have been shown to modulate cognitive function and synaptic plasticity (McEwen and Sapolsky, 1995; Diamond et al., 1996; Kim et al., 1996; Sandi et al., 1997; Cao et al., 2016). Long-term stress has also been shown to induced morphological changes in the hippocampus (Lee et al., 2009; Schoenfeld and Gould, 2012). In addition, hippocampus also important to anxiety (Barkus et al., 2010) and depression (Sahay and Hen, 2007). Therefor this study focused on hippocampus.

The level of corticosterone was assessed as a biomarker of stress in rodents, it was reported that chronic stress could induce plasma corticosterone increasing sustainedly (Bernatova et al., 2018). Sustained corticosterone rise is a key factor for stress-induced memory deficits (Dominguez et al., 2019). It was also reported that corticosterone itself could cause hippocampal synaptic plasticity and cognitive impairment (Pavlides et al., 1993; Howland and Wang, 2008). Therefore, corticosterone was adopted to mimic stress. Results showed that single administration of corticosterone *via* subcutaneous injection impaired hippocampal LTP in mice *in vivo* significantly, both intracerebroventricular and intragastric administrated of isorhynchophylline reversed corticosterone-induced LTP impairment, suggesting that isorhynchophylline may have protective effect against stress-induced synaptic and cognitive impairment. Previous study reported that intragastric administration of isorhynchophylline 20 mg/kg for 7 days had anti-depression effects (Xian et al., 2017), and 20 mg/kg isorhynchophylline was sufficient to reverse corticosterone-induced LTP impairment in this study. So 20 mg/kg isorhynchophylline was adopted for further observing its effect on emotional disorders and cognitive impairment caused by chronic stress.

Chronic unpredictable mild stress is widely used to investigate stress induced disorders, such as depression and cognitive impairment (Shang et al., 2017; Willner, 2017; Antoniuk et al., 2019). Therefore, CUMS was adopted in this study. Results showed that CUMS caused weight loss, in accord with previous study (Willner et al., 1996), and isorhynchophylline significantly alleviated CUMS-induced weight loss. The plasma corticosterone was assessed as a biomarker of stress, previous research showed that attenuating corticosterone on the day of memory assessment prevented chronic stress-induced spatial memory impairments (Wright et al., 2006), our results also showed that isorhynchophylline decreased elevated corticosterone in CUMS mice, indicating that isorhynchophylline may reduce stress response. SPT was used to evaluate depression-like behavior, results showed that CUMS caused depression-like behavior, in accord with previous reports (Qiao et al., 2016; Liu W. et al., 2018), and isorhynchophylline alleviated CUMS-induced depression-like behavior in line with reported results (Xian et al., 2017). For anxiety-like behavior, OPT and EPM were adopted. The results showed that isorhynchophylline significantly enhanced the exploratory behavior of CUMS mice, suggesting that the anxiety symptoms were efficiently relieved by isorhynchophylline. MWM test is the most commonly used experiment to evaluate spatial learning and memory (Vorhees and Williams, 2006). CUMS-induced cognitive impairment has been widely reported (Gu et al., 2014; Shen et al., 2018, 2019), our results also showed that CUMS caused spatial memory deficit in mice and isorhynchophylline reversed this impairment. These results indicates that isorhynchophylline improved both emotional disorder and cognitive impairment.

**FIGURE 6:**
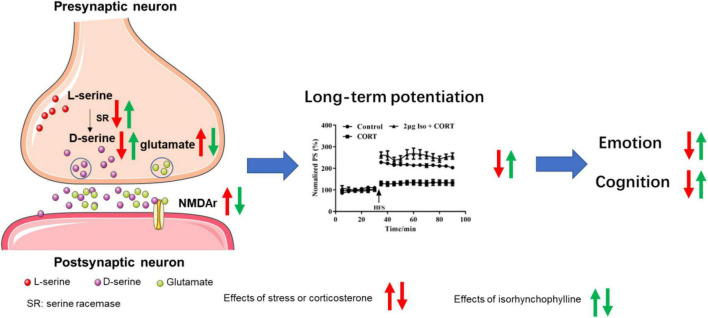
Sketch map for the mechanisms of isorhynchophylline on CUMS induced emotional disorders and cognitive impairment.

Synaptic structure is the biological basis of learning and memory (Luscher and Malenka, 2012), normal synaptic transmission requires presynaptic glutamate and D-serine to bind to NMDA receptors to maintain synaptic plasticity and cognitive function (Hardingham and Bading, 2010). It’s reported that chronic stress induced glutamate elevation (Garcia-Garcia et al., 2009; Hill et al., 2012; Willner, 2017) and NMDA receptors expression increasing (Liu et al., 2019; Lorigooini et al., 2020) in hippocampal tissue, indicating hyperfunction of NMDA receptors. On the other hand, chronic stress induced D-serine deficit (Wang et al., 2017), indicating hypofunction of NMDA receptors. Our previous studies provide possible explanation for this paradox. Previous data showed that acute stress or corticosterone administration may cause hypofunction of NMDA receptors by inhibiting D-serine release, despite of increasing glutamate (Wang et al., 2021). Long-term hypofunction of NMDA receptor, on the condition of chronic stress, might cause compensatory NMDA receptor expression, as showed in published data (Liu et al., 2019; Lorigooini et al., 2020) and this study. Isorhynchophylline improved LTP impairment by corticosterone *via* ICV administration, indicating isorhynchophylline improved corticosterone induced NMDA receptors hypofunction. Additionally, isorhynchophylline restored CUMS induced glutamate and D-serine disturbance, and modulated the expression of serine racemase (SR) and NMDA receptors. Altogether, these results indicate that modulating the function of NMDA receptor may be involved in the effect of isorhynchophylline on alleviating cognitive deficits induced by CUMS (Figure 6).

## 5 Conclusion

In conclusion, isorhynchophylline has protective effect against stress-induced emotional disorders and cognitive impairment simultaneously, restoring the function of NMDA receptors to normal might be one of its mechanisms. Nevertheless, the underlying mechanisms are more complex than it was described above and further investigations are needed.

## Data availability statement

The original contributions presented in this study are included in the article/supplementary material, further inquiries can be directed to the corresponding authors.

## Ethics statement

This animal study was reviewed and approved by the Institute of Animal Care and Use Committee (IACUC) of the National Beijing Center for Drug Safety Evaluation and Research (NBCDSER).

## Author contributions

YH, W-XZ, and Y-XZ conceived the study and participated in design. CW conducted most of the experiments. WS, M-HZ, and NJ conducted parts of the experiments. CW and YH wrote and revised the manuscript. W-XZ and Y-XZ revised the manuscript. NS participated in the analysis of the results of the electrophysiological experiment. Q-SZ provided isorhynchophylline used in this study. All authors contributed to the article and approved the submitted version.

## Footnotes

1
http://www.winltp.com
